# Molecular Characterization of *Streptococcus agalactiae* Causing Community- and Hospital-Acquired Infections in Shanghai, China

**DOI:** 10.3389/fmicb.2016.01308

**Published:** 2016-08-30

**Authors:** Haoqin Jiang, Mingliang Chen, Tianming Li, Hong Liu, Ye Gong, Min Li

**Affiliations:** ^1^Department of Laboratory Medicine, Shanghai Medical College, Huashan Hospital, Fudan UniversityShanghai, China; ^2^Shanghai Municipal Center for Disease Control and PreventionShanghai, China; ^3^Shanghai Institutes of Preventive MedicineShanghai, China; ^4^Department of Laboratory Medicine, School of Medicine, Renji Hospital, Shanghai Jiao Tong UniversityShanghai, China; ^5^Department of Critical Care Medicine, Shanghai Medical College, Huashan Hospital, Fudan UniversityShanghai, China

**Keywords:** *Streptococcus agalactiae*, antimicrobial susceptibility testing, serotype, multilocus sequence type (MLST), pulsed-field gel electrophoresis (PFGE), virulence factor

## Abstract

*Streptococcus agalactiae*, a colonizing agent in pregnant women and the main cause of neonatal sepsis and meningitis, has been increasingly associated with invasive disease in nonpregnant adults. We collected a total of 87 non-repetitive *S. agalactiae* isolates causing community-acquired (CA) and hospital-acquired (HA) infections in nonpregnant adults from a teaching hospital in Shanghai between 2009 and 2013. We identified and characterized their antibiotic resistance, sequence type (ST), serotype, virulence, and biofilm formation. The most frequent STs were ST19 (29.9%), ST23 (16.1%), ST12 (13.8%), and ST1 (12.6%). ST19 had significantly different distributions between CA- and HA-group B *Streptococci* (GBS) isolates. The most frequent serotypes were III (32.2%), Ia (26.4%), V (14.9%), Ib (13.8%), and II (5.7%). Serotype III/ST19 was significantly associated with levofloxacin resistance in all isoates. The HA-GBS multidrug resistant rate was much higher than that of CA-GBS. Virulence genes *pavA, cfb* were found in all isolates. Strong correlations exist between serotype Ib (CA and HA) and surface protein genes *spb1* and *bac*, serotype III (HA) and surface protein gene *cps* and GBS pilus cluster. The serotype, epidemic clone, PFGE-based genotype, and virulence gene are closely related between CA-GBS and HA-GBS, and certain serotypes and clone types were significantly associated with antibiotic resistance. However, CA-GBS and HA-GBS still had significant differences in their distribution of clone types, antibiotic resistance, and specific virulence genes, which may provide a basis for infection control.

## Introduction

*Streptococcus agalactiae* (group B *streptococcus* [GBS]), a common commensal of the female genital tract, is well established as a main cause of neonatal sepsis and meningitis and as the most common agent of invasive infections in pregnant women (Romanik et al., 2014). Nevertheless, in the past decade, GBS has been increasingly associated with invasive disease in nonpregnant adults (Murayama et al., 2009). Common presentations of GBS disease in adults include skin and/or soft-tissue infection, bacteremia without focus, pneumonia, urinary tract infections, and osteomyelitis; serious clinical syndromes, such as meningitis and endocarditis, are rare but often associated with considerable morbidity and mortality (Al et al., 2010; Le Doare and Heath, 2013). Although GBS infections are most frequently community-acquired (CA), hospital-acquired (HA) disease is also of concern (Martins et al., 2012; Le Doare and Heath, 2013).

Group B *Streptococci* has a variety of virulence factors that facilitate its ability to cause disease, some of which have been identified and characterized. Several virulence determinants are involved in the adhesion to and invasion of host cells, as well as in evasion from the immune system (Dutra et al., 2014). These include capsular polysaccharides (CPSs), regulatory proteins, surface-localized proteins, and toxins (Al et al., 2011). GBS makes use of a number of virulence factors, including pore-forming toxins that damage host cells, adhesion factors that increase binding to cells or to the extracellular matrix, evasion factors that decrease neutrophil recruitment and prevent complement binding, and factors that repel or otherwise induce resistance to antimicrobial peptides (Chen et al., 2013). CPSs are recognized as playing key roles as virulence factors and are important targets for the development of vaccine strategies (Bellais et al., 2012; Chen et al., 2013). GBS CPSs have chemical and antigenic differences that enable the subdivision of this species into 10 CPS serotypes (Ia, Ib, II, III, IV, V, VI, VII, VIII, and IX) (Beigverdi et al., 2014; Dutra et al., 2014), dominated by five CPS types (Ia, Ib, II, III, and V) (Stoner et al., 2015), which account for 96 and 88% of cases of invasive GBS infections in neonates and adults, respectively (Bellais et al., 2012; Dutra et al., 2014). The available data suggest that the serotype distribution of GBS isolates is similar in Africa, the Western Pacific, Europe, the Americas, and the Eastern Mediterranean regions. Therefore, conjugate vaccines that include some or all of these serotypes hold great promise for preventing this important disease (Le Doare and Heath, 2013).

In recent years, the ability of GBS to form biofilms has attracted attention for its possible role in the fitness and virulence of this pathogen (D'Urzo et al., 2014). Interestingly, GBS human isolates can form biofilms on both abiotic and biotic surfaces (Rinaudo et al., 2010). Several studies (Rinaudo et al., 2010; D'Urzo et al., 2014) have shown that biofilm formation was a barrier that protected the pathogen against antibiotics and human immune system pressure, and it restricted the diffusion of antibiotics, lessening the need for the expression of genes that would allow the cells to tolerate these substances.

Maternal and neonatal populations are those most commonly infected by GBS, and previous reports have generally focused on these special populations; however, little is known about the molecular epidemiology of the *S. agalactiae* isolates that cause CA and HA infections in nonpregnant adults. Therefore, this study was conducted to investigate and characterize the antibiotic resistance, sequence type (ST), serotype, virulence, and biofilm formation of GBS isolates and to compare the clinical feathers of CA- and HA-GBS, which may provide a useful basis for infection control.

## Materials and methods

### Clinical definitions

CA *S. agalactiae* (CA-GBS) was defined as an isolate that was obtained either from an outpatient or from an inpatient ≤ 48 h after hospital admission and lacking the following risk factors: contact with the hospital environment in the 6 months preceding the culture, *S. agalactiae* infection history, residence in a long-term care facility in the 12 months preceding the culture. HA *S. agalactiae* (HA-GBS) was defined as an isolate that was obtained from an inpatient >48 h after hospital admission or having at least one of the risk factors decribed above (Chuang and Huang, 2013; Horcajada et al., 2013). The definition of infection followed the guidelines published by the Centers for Disease Control and Prevention (CDC) (Stevenson et al., 2005; Maree et al., 2007; CDC, 2016a,b).

### Ethics statement

This study was approved by the ethics committee of Huashan Hospital, Shanghai Medical College, Fudan University, Shanghai, People's Republic of China (protocol HS-H-2014-0213). All subjects provided written informed consent before their inclusion in the study.

### Bacterial isolates

In this study, a total of 87 unique *S. agalactiae* isolates causing CA (42.5%) and HA (57.5%) infections in nonpregnant adults (all patients were ≥18 years old) were collected from Shanghai teaching hospital from 2009 to 2013. *S. agalactiae* isolates were isolated from urine (55 isolates), respiratory samples (15 isolates), skin/soft tissue (10 isolates), vaginal fluid (5 isolates), and drainage fluid (2 isolates). Almost 58 (66.6%) of the isolates were from women and 29 (33.4%) were from men. Two subpopulations were considered, the younger was aged from 18 to 64 (73.5%), and the elderly ≥65 years old (26.5%). Bacteria were identified as *S. agalactiae* by Gram staining, colony morphology, and detection of β-hemolysis and were further characterized using VITEK® 2 Compact GP ID Card (bioMérieux, Marcy l'Etoile, France). *S. agalactiae* isolates were cultured in brain heart infusion medium broth (BHI) (OXOID, Basingstoke, Britain) at 37°C with 5% CO_2_, unless specified otherwise.

### Antimicrobial susceptibility testing

Susceptibility to penicillin G, vancomycin, erythromycin, clindamycin, levofloxacin, cefprozi, ceftriaxone, cefotaxime, and linezolid was measured by performing Kirby–Bauer's disk diffusion (OXOID, Basingstoke, Britain) according to the Clinical and Laboratory Standard Institute (CLSI) standards (CLSI, 2014). *S. pneumoniae* ATCC 49619 was used as a control strain. Multidrug resistance (MDR) was defined as resistance to more than any three antimicrobial agents of different classes tested in this study (Zhang et al., 2013).

### Serotyping

*Streptococcus agalactiae* isolates were cultured in Todd-Hewitt liquid medium (TH; OXOID, Basingstoke, Britain). Capsular serotyping of all isolates was carried out by a latex agglutination assay with a Group-B *Streptococci* Typing Antisera kit (Denka Seiken, Japan) according to the manufacturer's instructions.

### Pulsed-field gel electrophoresis profiling and multilocus sequence type

Genomic DNA was extracted from bacterial culture using EZ-10 spincolumn DNA isolation kits (Sangon, Shanghai, China) according to the manufacturer's instructions. Pulsed-field gel electrophoresis (PFGE) was performed for all isolates after SmaI macrorestriction, as previously described by Udo et al. (2013). PFGE was used to compare the genetic diversity of the dominant STs recovered from the same ward. Briefly, SmaI-digested DNA embedded in agarose plugs was subjected to PFGE analysis at 14°C in a CHEF-MAPPER system (Bio-Rad) for 20 h at 6.0 V/cm, in 0.5 × Tris-borate-EDTA buffer for one stage consisting of a 10 s initial pulse and a 45 s final pulse at a 120° angle. Analysis of the digitized images was assisted by the software BioNumerics (Applied Maths, Belgium), and each different strip was considered to be a different PFGE type (Tenover et al., 1995). A dendrogram was constructed by using unweighted pair group with arithmetic averages (UPGM) according to the similarity coefficient. PFGE-based clusters were defined as groups of two or more isolates with a dice coefficient of ≥80% on the dendrogram (Martins et al., 2012). Determination of multilocus sequence type (MLST) was performed by sequencing seven housekeeping genes (*adhP, phes, atr, gInA, sdhA, glcK*, and *tkt*), and ST assignment was determined by analyzing the entire *S. agalactiae* MLST database (http://pubmlst.org/sagalactiae) and using goeBURST V3. Analysis of DNA sequences was performed by using DNASTAR Seqman Pro software (DNASTAR, Inc. Madison, USA).

### Semi quantitative biofilm formation test

Biofilm formation was tested using BHI culture-treated polystyrene 96-well flat plates according to the procedure reported by Wang et al. (2008) with the following modifications. After the cells were fixed in Bouin's fixative for 1 h, the cells were washed gently four times in phosphate-buffered saline and then stained with 0.1% crystal violet solution. The stain was washed off gently under slowly running water, and the plates were dried at room temperature. The absorbance of the stained biofilm was measured at 570 nm using a MicroELISA autoreader (Bio-Rad).

### Virulence genes

A total of 45 major GBS virulence genes (Rosenau et al., 2007; Smith et al., 2007; Ding et al., 2009; Lin et al., 2011; Hanson et al., 2012; Firon et al., 2013; Rato et al., 2013) were detected, and amplification was carried out on a GeneAmp 9700 thermal cycler (Applied Biosystems, NY, USA) under the following conditions: an initial 5 min denaturation at 94°C, followed by 30 cycles of 30 s at 94°C, 30 s at 52°C, and 30 s at 72°C, with a final extension at 72°C for 10 min (Smith et al., 2007; Lin et al., 2011). The sequences of primers used to identify putative and known GBS virulence genes with systematic gene names are shown in Table S1. The PCR fragments were visualized by agarose gel electrophoresis and ethidium bromide staining.

### Typing concordance and statistical analysis

For statistical analysis, the Wallace coefficient (W) provides a quantitative measure of the clustering concordance between different typing methods (Pinto et al., 2008; Martins et al., 2012). In our collection, the Wallace coefficient was calculated to determine the concordance between PFGE-based clustering, serotyping, STs, antimicrobial resistance and virulence gene profiling. Simpson's index of diversity (SID) was used to evaluate the diversity found among the isolates studied (Carrico et al., 2006; Martins et al., 2012). Both these calculations, as well as the 95% confidence intervals (CI95%) were performed with the Web tools available at http://www.comparingpartitions.info. The Fisher exact test, as appropriate, was used to evaluate the significance of associations. The odds ratio (OR) with 95% Wald confidence intervals (CI95%) was calculated against all other serotypes, STs or PFGE clusters and used to identify particular serotypes, STs or PFGE clusters associated with certain characteristics, controlling for a false-discovery rate (FDR) less than or equal to 0.05 (Martins et al., 2012; Glickman et al., 2014).

## Results

### Serotype and MLST distribution among PFGE clusters

**Figure 1:**
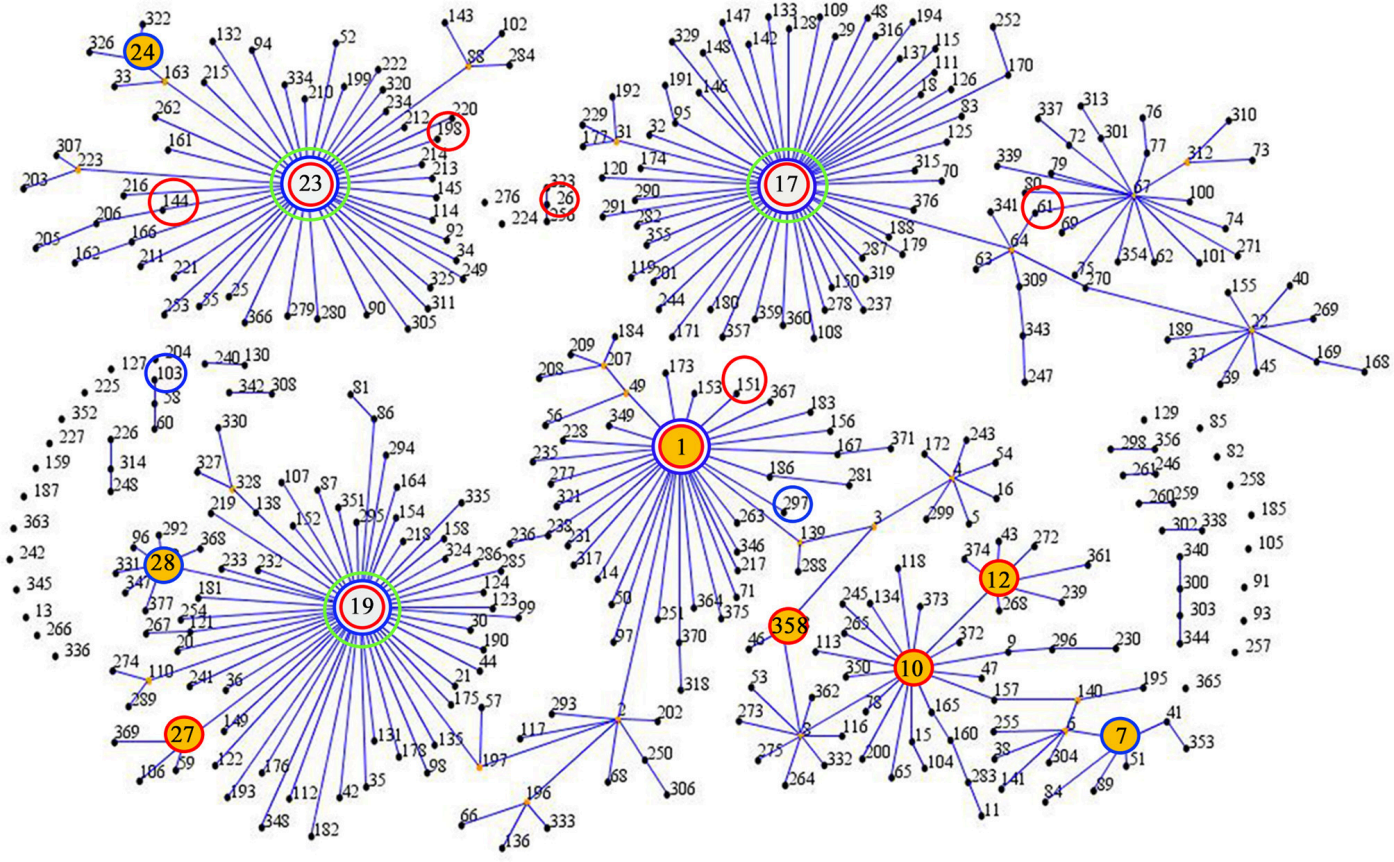
**eBURST analysis of ***S. agalactiae*** using all STs available in the MLST database as of January 2016**. ST nodes: green circles indicate a group founder, yellow circles indicate a sub-group founder, red circles indicate STs isolated from community samples, and blue circles indicate STs isolated from hospital samples.

Twenty-two different STs were identified in the 87 collected GBS isolates, the predominant STs of the isolates were identified as ST19 (29.9%), ST23 (16.1%), ST12 (13.8%), and ST1 (12.6%). The results of STs were summarized in Table 1, 17 different STs were identified in CA-GBS isolates, reflecting considerable STs diversity (SID 0.922; CI_95%_, 0.884 to 0.960), but only 10 STs were identified in HA-GBS isolates, reflecting considerable STs diversity (SID 0.789; CI_95%_, 0.705 to 0.872). The difference between the distribution of ST19 in CA-GBS isolates and that in HA-GBS isolates was statistically significant (Fisher's exact test, *p* = 0.0191). The homology of the 87 GBS strains was analyzed using an eBURST V3 analysis (Figure 1), which identified eight main clonal complexes (CCs) and four singleton STs that were not identified as part of a clonal complex (Table 1). Additionally, we also found one new STs: *adhP* 9, *phes* 5, *atr* 4, *gInA* 1, *sdhA* 3, *glck* 3, and *tkt* 2.

**Table 1:** **Serotypes and antimicrobial susceptibility profiles of ***S.agalactiae*** isolates arranged by MLST (STs) and clonal clusters (CCs)**.

**STs**	**CCs**	**Acquired^a^**	**No.^b^**	**Serotypes**							**Biofilm%**	**Erythromycin**	**Clindamycin**	**Levofloxacin**	**Cefprozi**	**Ceftriaxone**	**Cefotaxime**	**Linezolid**
				**Ia**	**Ib**	**II**	**III**	**IV**	**V**	**NT^c^**		**Antimicrobial agent^d^ %Resistance^e^**						
ST-19	CC-19	CA	6	0	0	0	6	0	0	0	0	50.0	16.7^**^	100.0^***^	0.0	0.0	0.0	0.0
		HA	20	0	0	0	18	0	1	1	1 (5.0)	70.0	65.0^**^	70.0^***^	5.0	5.0	0.0	5.0
ST-23	CC-23	CA	5	5	0	0	0	0	0	0	1 (20.0)	60.0	0.0^**^	0.0	0.0	0.0	0.0	0.0
		HA	9	8	0	0	1	0	0	0	2 (22.2)	77.8	22.2^*^	11.1	0.0	22.2	0.0	0.0
ST-12	CC-10	CA	6	0	4	2	0	0	0	0	4 (66.7)	100.0	100.0	0.0	0.0	0.0	0.0	0.0
		HA	6	0	5	1	0	0	0	0	0	100.0	100.0	0.0	0.0	16.7	8.3	8.3
ST-1	CC-1	CA	5	0	0	1	0	0	4	0	0	100.0	100.0	0.0	0.0	0.0	0.0	0.0
		HA	6	0	0	0	0	0	5	1	0	83.3	83.3	0.0	16.7	16.7	0.0	0.0
ST-485	–	CA	2	1	0	0	0	0	0	1	1 (50.0)	1	0	0	0	0	0	0
		HA	1	0	1	0	0	0	0	0	0	0	0	0	0	0	0	0
ST-7	CC-7	HA	2	2	0	0	0	0	0	0	1 (50.0)	0	0	0	0	0	0	1
ST-24	CC-24	HA	2	2	0	0	0	0	0	0	1 (50.0)	0	0	0	0	0	0	0
ST-103	–	HA	2	2	0	0	0	0	0	0	0	1	0	0	0	0	0	0
ST-198	CC-23	CA	2	2	0	0	0	0	0	0	0	1	0	0	0	0	0	0
ST-10	CC-10	CA	1	0	1	0	0	0	0	0	1	1	1	1	0	0	0	0
ST-17	CC-17	CA	1	0	0	0	1	0	0	0	0	1	1	0	0	0	0	0
ST-26	–	CA	1	0	0	0	0	0	1	0	0	0	0	0	0	0	0	0
ST-27	CC-19	CA	1	0	0	0	1	0	0	0	0	1	0	0	0	0	0	0
ST-28	CC-19	HA	1	0	0	0	0	0	0	1	0	1	1	0	0	0	0	0
ST-61	CC-67	CA	1	0	0	0	1	0	0	0	0	0	0	0	0	0	0	0
ST-144	CC-23	CA	1	1	0	0	0	0	0	0	0	1	1	0	0	0	0	0
ST-151	CC-1	CA	1	0	0	0	0	0	1	0	0	0	0	0	0	0	0	0
ST-297	CC-1	HA	1	0	0	0	0	0	0	1	0	0	0	0	0	0	0	0
ST-358	CC-10	CA	1	0	1	0	0	0	0	0	0	1	1	0	0	0	0	0
ST-591	–	CA	1	0	0	0	0	0	1	0	0	1	0	0	0	0	0	0
ST-652	CC-10	CA	1	0	0	0	0	0	0	1	0	0	0	0	0	0	0	0
ST-new	CC-10	CA	1	0	0	1	0	0	0	0	0	1	1	0	0	0	0	0
**Total (%)**			**87**	**26.4**	**13.8**	**5.7**	**32.2**	**0.0**	**14.9**	**6.9**	**13.8**	**69.0**	**50.6**	**25.3**	**2.3**	**8.0**	**1.1**	**2.3**

a *CA, community-acquired infection; HA, hospital-acquired infection*.

b *STs with less than five isolates were not calculated in the percentage of antibiotic resistance*.

c *NT, non-typeable*.

d *All strains were susceptible to penicillin and vancomycin*.

e *Boldface indicates that serotype III/ST19 and serotype Ia/ST23 were significantly associated with certain antimicrobial resistance in the CA-GBS and HA-GBS isolates. Boldface and italic indicates that serotype III/ST19 showed significantly different resistance rate between CA-GBS and HA-GBS isolates*.

* *p < 0.05*,

** *p < 0.01*,

*** *p < 0.001*.

Of the 87 total collected GBS strains, the main sertotypes were serotypes III (32.2%), Ia (26.4%), V (14.9%), Ib (13.8%), and II (5.7%). The results of serotyping were summarized in Table 1, reflecting considerable overall serotype diversity (CA: SID 0.827; CI_95%_, 0.790 to 0.865, HA: SID 0.757; CI_95%_, 0.690 to 0.824, respectively). There was no significant difference in the serotype distribution between CA-GBS and HA-GBS isolates (Fisher's exact test, all *p* > 0.05). These five predominant serotypes accounted for 93% of all the tested GBS strains. However, some of isolates were non-typeable (NT) as a result of a limitation of the reagent. Notably, we did not find any serotype IV isolates in our research. Interestingly, we found that the concordance between STs and the serotype, as given by W_STs → *serotype*_ = 0.796 (CI_95%_, 0.669–0.923), showed that 79.6% of any pair of isolates in the same STs also share the same serotype. The certain STs were significantly associated with particular serotypes both in CA- and HA-GBS, namely, ST19 and serotype III, ST23 and serotype Ia, ST12 and serotype Ib, and ST1 and serotype V (Table 1).

**Figure 2:**
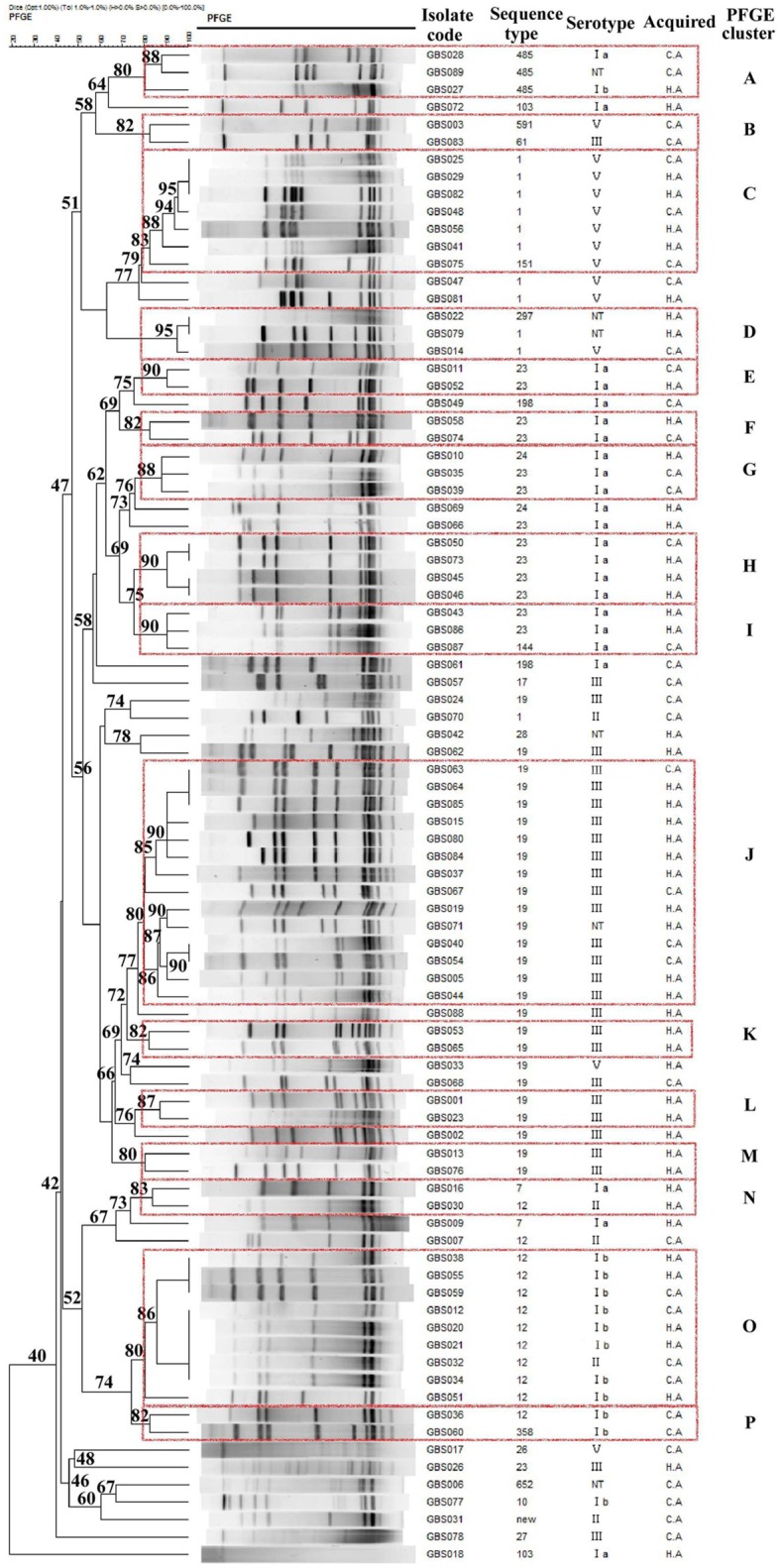
**Dendrogram of the PFGE profiles of 87 GBS isolates**. The dendrogram was constructed using the UPGMA method. Dice coefficients (percentages) are indicated in the scale above the dendrogram. Each cluster (defined as a group of two or more isolates with a Dice coefficient of ≥80%) is represented in the dendrogram.

The isolates in this study were grouped in 16 PFGE clusters (represented by dashed rectangles in Figure 2) according to 80% similarity on the dendrogram, each including 2–14 strains. The remaining isolates (*n* = 24) had unique profiles. The SID for the classification of the CA and HA isolates in PFGE clusters was 0.971 (CI_95%_, 0.950–0.993), and 0.943 (CI_95%_, 0.905–0.981), respectively, indicating that the collection analyzed was quite diverse and there was no significant difference in the PFGE-based genotype distribution between CA-GBS and HA-GBS isolates (Fisher's exact test, all *p* > 0.05). The concordance between PFGE-based genotypes and the serotype, as given by W_PFGE→serotype_ = 0.839 (CI_95%_, 0.696 to 0.982), showed that 83.9% of any pair of isolates in the same PFGE also share the same serotype. The concordance between PFGE-based genotypes and the eBURST-based genotypes, as given by W_PFGE→sBURST_ = 0.960 (CI_95%_, 0.943–0.976), showed that 96.0% of any pair of isolates in the same PFGE also share the same CCs. The certain PFGE-based genotypes were significantly associated with particular CCs both in CA- and HA-GBS (Figure 2), showing that cluster A, C, and D belonged to CC1, clusters E, F, G, H, and I belonged to CC23, Clusters J, K, L, and M belonged to CC19, Clusters N, O, and P belonged to CC10, respectively. We observed that the identification of different GBS strains by PFGE provides higher resolution, which could fully reflect the gene polymorphisms in these isolates.

### Antimicrobial resistance

All tested isolates including CA- and HA-GBS were sensitive to both penicillin and vancomycin and showed high rates of resistance to erythromycin (69.0%) and clindamycin (50.6%), especially CA-ST12 and HA-ST12 (100%). The overall percentages of levofloxacin resistance was 23.5% while cefprozil, ceftriaxone, cefotaxime, and linezolid resistance were much lower, at 2.3, 8.0, 1.1, and 2.3%, respectively. Both CA-GBS and HA-GBS isolates of serotype III/ST19 were significantly associated with levofloxacin resistance. Notably, the rate of levofloxacin resistance both in CA- and HA-GBS was significantly higher in serotype III than in serotypes Ia, Ib, II, and V. Moreover, HA-serotype III/ST19 showed higher resistance rates to clindamycin than CA-serotype III/ST19. Additioanlly, the rate of clindamycin resistance in both CA- and HA-GBS was significantly lower in serotype Ia/ST23. Notably, no significant differences were found in the rates of resistance to erythromycin, clindamycin, or levofloxacin among the other STs or serotypes (in either CA-GBS or HA-GBS). Lastly, we found that the HA-GBS MDR rate was much higher than the MDR rate of CA-GBS isolates (OR = 9.844; CI_95%_; 2.115–45.82). The overall MDR rate for all 87 of the collected GBS isolates was 23.0% (20/87; Table 1).

### Biofilm formation

Only 12 (13.8%) isolates produced positive results from the biofilm formation test. Biofilm formation was not associated with the antibiotic resistance, serotype, or clone type of either CA-GBS or HA-GBS isolates. The erythromycin resistance rate was 83.3% (10/12) in biofilm-positive strains, but the ability to form a biofilm was not associated with resistance to other antibiotics, such as erythromycin and clindamycin.

### Distributional characteristics of virulence-associated factors

Fourty-five GBS virulence genes that were related to adhesion, invasion, or immune evasion were detected in our isolates (Table 2). Virulence genes *pavA, cfb, neuC*, and *pbp1A* were found in all GBS isolates, but the *cpsJ* gene was not found in any of the isolates. We also found a strong correlation of particular genes encoding virulence proteins with certain serotypes (Table 3).

**Table 2:** **Distribution of virulence genes encoding particular proteins between CA-GBS and HA-GBS**.

**Category**	**Virulence gene^a^**	**Total no. of isolates with virulence gene^b^ (%)**		
		**CA**	**HA**	**Total**
Adhesion	*fbsA*	29 (33.3)	33 (37.9)	62 (71.3)
	*fbsB*	34 (39.1)	49 (56.3)	83 (95.4)
	*scpB*	34 (39.1)	47 (54.0)	81 (93.1)
	*lmb*	32 (36.8)	47 (54.0)	79 (90.8)
	*gbs 0628*^c^	23 (26.4)	36 (41.4)	59 (67.8)
	*gbs 0629*^c^	22 (25.3)	38 (43.7)	60 (69.0)
	*gbs 0630*^c^	23 (26.4)	35 (40.2)	58 (66.7)
	*gbs 0631*^c^	24 (27.6)	37 (42.5)	61 (70.1)
	*gbs 0632*^c^	23 (26.4)	36 (41.4)	59 (67.8)
Invasion	*cylX*	36 (41.4)	44 (50.6)	80 (92.0)
	*cylD*	35 (40.2)	46 (52.9)	81 (93.1)
	*cylG*	36 (41.4)	45 (51.7)	81 (93.1)
	*acpC*	36 (41.4)	47 (54.0)	83 (95.4)
	*cylZ*	37^*^ **(42.5)**	43^*^ **(49.4)**	80 (92.0)
	*cylA*	37^*^ **(42.5)**	42^*^ **(48.3)**	79 (90.8)
	*cylB*	37 (42.5)	47 (54.0)	84 (96.6)
	*cylE*	36 (41.4)	44 (50.6)	80 (92.0)
	*cylF*	34 (39.1)	45 (51.7)	79 (90.8)
	*cylI*	37 (42.5)	49 (56.3)	86 (98.9)
	*cylJ*	36 (41.4)	48 (55.2)	84 (96.6)
	*cylK*	8 (9.2)	8 (9.2)	16 (18.4)
	*spb1*	14 (16.1)	13 (14.9)	27 (31.0)
	*hylB*	7 (8.0)	9 (10.3)	16 (18.4)
	*rib*	4 (4.6)	3 (3.4)	7 (8.0)
	*bca*	22 (25.3)	31 (35.6)	53 (60.9)
Immune evasion	*bac*	11 (12.6)	10 (11.5)	21 (24.1)
	*cpsM*	22 (25.3)	33 (37.9)	55 (63.2)
	*cpsIaJ*	19 (21.8)	33 (37.9)	52 (59.8)
	*cpsI*	9 (10.3)	18 (20.7)	27 (31.0)
	*cpsG*	7 (8.0)	18 (20.7)	25 (28.7)
	*cpsF*	4 (4.6)	10 (11.5)	14 (16.1)
	*cpsE*	9 (10.3)	13 (14.9)	22 (25.3)
	*cpsD*	23 (26.4)	34 (39.1)	57 (65.5)
	*cpsC*	30 (34.5)	43 (49.4)	73 (83.9)
	*cpsB*	28 (32.2)	38 (43.7)	66 (75.9)
	*cpsA*	10 (11.5)	12 (13.8)	22 (25.3)
	*neuA*	18 (20.7)	26 (29.9)	44 (50.6)
	*neuD*	22 (25.3)	29 (33.3)	51 (58.6)
	*neuB*	30 (34.5)	38 (43.7)	68 (78.2)
	*cspA*	36 (41.4)	50 (57.5)	86 (98.9)
Total		37 (42.5)	50 (57.5)	87 (100.0)

a *All strains were positive for pavA, cfb, neuC, and pbp1A and were negative for cpsJ*.

b *Boldface indicates that there was a significantly different distributions in the CA-GBS and HA-GBS isolates*.

* *p < 0.05*.

c *GBS pilus cluster*.

**Table 3:**
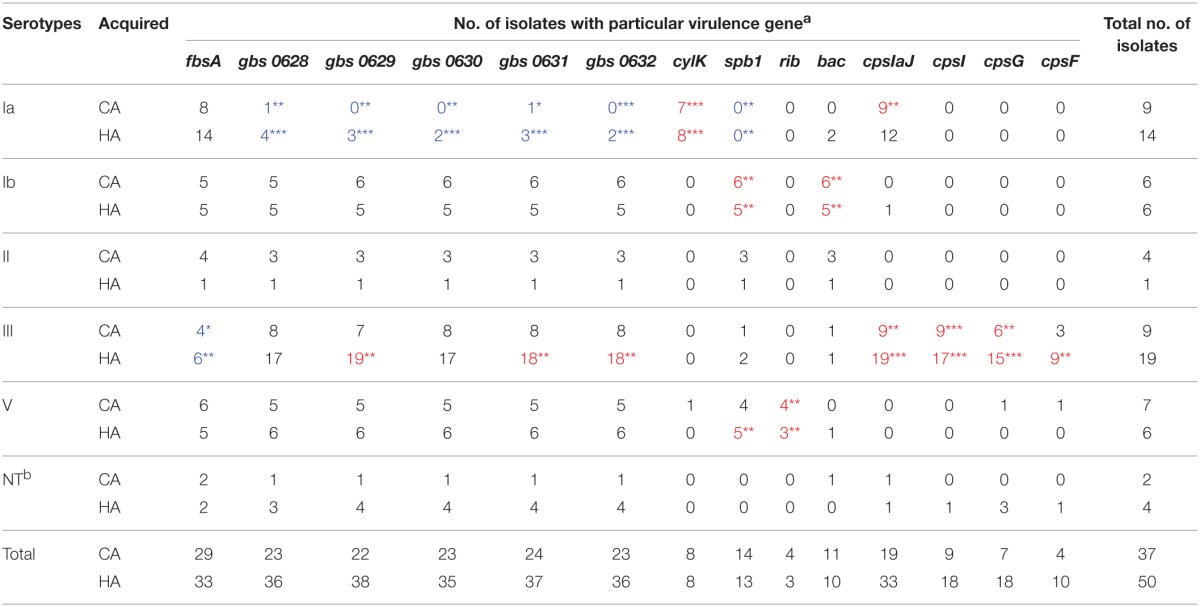
**Correlation between GBS particular virulence gene and serotypes across source of the infections**.

^a^Red and boldface indicates that there was a significant positive correlation between particular virulence gene and the serotype was found; blue and boldface indicates that there was a significant negative correlation between particular virulence gene and the serotype was found. ^*^p < 0.05, ^**^p < 0.01, ^***^p < 0.001.

*^b^NT, non-typeable*.

#### Adhesion-related genes

There was a significant negative association between particular adhesion-related genes and certain serotypes (CA and/or HA), namely, between *fbsA* and serotype III (CA and HA), and between GBS pilus cluster and serotype Ia (CA and HA). Additionally, there was also a significant positive association between particular adhesion-related genes in the GBS pilus cluster and certain serotypes (CA and/or HA), specifically serotype III (HA). GBS pilus cluster genes were found in serotype Ia (CA and HA) isolates less often than they were detected in other serotypes (Table 3).

#### Invasion-related genes

Most β-hemolysin/cytolysin (cyl) genes could be found in all of the isolates; however, *cylK* (18.4%) and *spb1, hylB*, and *rib* could only be found in a few CA and HA isolates. There was a significant negative association between an invasion-related gene, *spb1* and serotype Ia (CA and HA). Additionally, there was a significant positive association between particular invasion-related genes and certain serotypes (CA and/or HA), namely, between *cylK* and serotype Ia (CA and HA), between *spb1* and serotype Ib (CA and HA), serotype V (HA), and between *rib* and serotype V (CA and HA). The invasion-related genes *cylA* and *cylZ* had significantly different distributions in the CA-GBS and HA-GBS isolates (Table 2).

#### Immune evasion-related genes

*CpsA, cpsE, cpsG*, and *cpsF*, all of which belong to the cps gene cluster, could only be found in a few CA and HA isolates. There was a significant positive association between particular immune evasion-related genes and certain serotypes (CA and/or HA), namely, between *bac* and serotype Ib (CA and HA), between *cpsIaJ* and serotype Ia (CA), serotype III (CA and HA), between *cpsI* and serotype III (CA and HA), between *cpsG* and serotype III (CA and HA), between *cpsF* and serotype III (HA) (Table 3).

## Discussion

In our study, certain serotypes and clone types were significantly associated with antibiotic resistance to drugs, such as clindamycin or levofloxacin, and significant differences were found in the resistance rates among the five serotypes in CA-GBS and HA-GBS. There was good consistency of CA-GBS and HA-GBS among STs, serotypes, and PFGE-based genotypes. Furthermore, there were strong correlations of the CA- and/or HA-serotypes with the genes encoding virulence proteins.

The most frequent STs were ST19, ST23, ST12, and ST1. ST19 had significantly different distributions in CA-GBS and HA-GBS isolates, while other STs had similar distributions between these groups. Previous work demonstrated that GBS strains of different serotypes differ in their abilities to associate with host cells; however, such studies selected strains on the basis of cps type rather than ST (Korir et al., 2014). A very conserved and consistent serotype distribution was observed among the GBS isolates causing neonatal invasive disease in Europe, including Portugal, Italy, Poland, and Greece, where capsular types Ia, V, and III were predominant (Gherardi et al., 2007; Martins et al., 2012; Liakopoulos et al., 2014; Romanik et al., 2014). However, in the GBS isolates causing invasive disease in nonpregnant adults in China, we observed that the dominant serotypes are noticeably different from those found in these countries. Our data showed that serotype III was the most prevalent in our study population in China both in CA-GBS and HA-GBS, followed by serotypes Ia, V, II, and Ib. These findings are similar to previous reports (Ferrieri et al., 2013; Wang et al., 2013) from Europe, including the United Kingdom, France, and Germany, and from North America (Al et al., 2010; Ferrieri et al., 2013; Kulkarni et al., 2013; Lamagni et al., 2013; Wang et al., 2013).

There was good consistency between certain STs and serotypes in CA-GBS and HA-GBS isolates, such as ST19 and serotype III, ST23 and serotype Ia, ST12 and serotype Ib, and ST1 and serotype V, which agrees with the findings of previous reports (Martins et al., 2012; Dutra et al., 2014). It has also been reported (Wang et al., 2013) that ST19 is associated with serotypes Ib, II, III, and IV, but we found mostly serotype III strains, along with a few serotype V strains in our research, and this is likely the reason that ST19 was mostly associated with serotype III in our study. The isolates belonging to the same clonal groups and/or serotypes were clustered in the same or approaching PFGE clusters. Although the identification of various GBS strains by PFGE provided high resolution, which may fully reflect the gene polymorphisms of these strains, there was no significant difference in the PFGE-based genotype distribution between CA-GBS and HA-GBS isolates.

Penicillin was originally the first-line drug used in the treatment and prophylaxis of GBS infections. The current alternatives for patients allergic to penicillin include macrolides and lincosamides (De Francesco et al., 2012). In our research in Shanghai, rates of CA-GBS and HA-GBS resistance to erythromycin, clindamycin, and levofloxacin were lower than those reported in Beijing (Wang et al., 2013). The incidence of macrolide resistance is relatively low in western countries, ranging from 14.5 to 32% (Gherardi et al., 2007; Phares et al., 2008; Murayama et al., 2009; De Francesco et al., 2012; Lamagni et al., 2013; Frohlicher et al., 2014). Although we are now more and more seriously about the normalize use of antibiotics both to outpatient and hospitalized patients, the high resistance was associated with antibiotic misuse previously. Furthermore, in our research, although there were no significant differences in the rates of erythromycin, clindamycin, or levofloxacin resistance between CA-GBS and HA-GBS isolates, HA-GBS MDR rate was much higher than that of CA-GBS isolates, which creates some difficulties during the clinical treatment of these infections and suggests that patients with HA-GBS infection should be considered different from patients with CA-GBS infection to avoid treatment failure.

Previous work has indicated that serotype V and ST1 are associated with erythromycin resistance (De Francesco et al., 2012; Martins et al., 2012; Frohlicher et al., 2014). Most of the CA- and HA-GBS isolates in our research showed high rates of resistance to erythromycin, and the serotype and molecular typing by MLST or PFGE showed that resistance to erythromycin was associated with a variety of serotypes/clones, especially serotype Ib/ST12. These findings indicate that both CA- and HA-GBS isolates have a variety of phenotypic and genotypic characteristics, and they suggest that macrolide-resistant isolates may arise by clonal spread. Given that 0.7–10.0% of patients are allergic to penicillin and that GBS strains have high rates of resistance to erythromycin and clindamycin, fluoroquinolones are a good alternative antibiotic for treating infections with GBS (Wang et al., 2013). The rates of clindamycin and levofloxacin resistance among the other four serotypes differed significantly in CA- and HA-GBS, such as the rate of levofloxacin resistance was significantly higher in serotype III isolates than in serotype Ia, Ib, II, or V isolates. Therefore, serotype III was associated with levofloxacin resistance. These findings are similar to those of previous reports (Ueno et al., 2012; Wang et al., 2013; Morozumi et al., 2014). Moreover, we found that ST19 (both CA-GBS and HA-GBS) was significantly associated with levofloxacin resistance and the rate of clindamycin resistance was significantly lower in CA- and HA-serotype Ia/ST23 than in other serotypes/clonotypes, even though it was lowest in serotypes II or Ib in other reports (Ueno et al., 2012; Wang et al., 2013), which may be due to regional and/or population differences between studies and as some serotypes have unique epidemiological features that may lead to invasive diseases and might occur in specific populations or geographic areas or be associated with antibiotic resistance (Dutra et al., 2014). Our research could provide a basis for using certain antibiotics against particular serotypes or STs of CA-GBS and HA-GBS.

Invasion-related genes *cylA* and *cylZ* had significantly different distributions in CA-GBS and HA-GBS isolates, while adhesion and immune evasion-related genes had similar distributions between these groups. The positive rates of virulence genes *fbsA, scpB, lmb, rib, bca, bac*, and *cfb* were similar to those reported by previous studies (Al et al., 2010, 2011; Udo et al., 2013; Dutra et al., 2014; Korir et al., 2014) except for *fbsB, hylB*, and *spb1*, which is likely due to the regional differences between our study and theirs (Al et al., 2011; Udo et al., 2013; Korir et al., 2014). Moreover, this study is the first to report the positive rates of GBS virulence genes, such as GBS pilus cluster, *cyl* operon, *cps* gene cluster, and *neu* gene cluster. In the last decade, a pilus-like structure was identified in GBS and shown to play an important role in the adhesion to and invasion of host cells, in biofilm formation, and in resistance to phagocyte killing (Firon et al., 2013). The β-hemolysin/cytolysin expressed by GBS is an important virulence factor encoded within a cluster of 12 genes forming the *cyl* operon, which is toxic to a broad range of eukaryotic cells, resulting in cell invasion and evasion of phagocytosis (Firon et al., 2013).

We also found a strong correlation of certain CA- and HA-serotypes with the genes encoding virulence proteins, such as between serotype Ib and *bac*, which was in agreement with the previous reports (Martins et al., 2012). However, we also found that of different findings, such as between serotypes V and *rib*, which was reported that of between serotypes II and III and *rib* (Martins et al., 2012). Moreover, we found that CA- and/or HA-serotype III/ST19 isolates were significantly associated with a few adhesion and immune evasion-related genes and that adhesion-related genes (*GBS pilus cluster*) and invasion-related genes (*spb1*) were expressed significantly less in CA- and HA-serotype Ia/ST23 isolates than in other serotypes/clones. A previous study reported that both ST17 and ST19 strains were more often associated with invasive disease but that ST23 strains were linked to asymptomatic colonization (Korir et al., 2014), which may explain our findings in this study. Certainly, not all isolates with particular serotypes and STs were associated with these genes, and the identification of virulence protein genes proved helpful in discriminating between different genetic lineages within serotypes. These studies have furthered appreciation of the roles that different serotypes and genotypes expressing different virulence factors play in CA- and HA-GBS diseases. Further research into these virulence genes may aid in the future development of new vaccines to reduce GBS infections.

There were still some limitations of the methods in our research. One was the limit number of the non-repetitive strains, which was not a large one and might lead to some statistical constraints although we had already made the adjustment to controlling for a false-discovery rate (FDR). The other was the limited reagent kit to identify GBS serotype, which was capable of only six serotypes including Ia, Ib, II, III, IV, and V, therefore uncovered serotypes could be think as NT.

In summary, CA-GBS and HA-GBS isolates had significantly similar correlations among STs, clonal clusters, PFGE types, serotypes, and virulence genes, but also had some differences in distribution of clonal, serotypes, virulence genes, and antimicrobial resistance, which show the molecular characterization. Most previous studies have focused on maternal and neonatal GBS infections; to our knowledge, our study is the first to report the GBS infection status of nonpregnant adults in China and is also the first to compare the molecular characteristics of HA-GBS and CA-GBS infection. In the near future, additional GBS strains should be collected from areas throughout China and further epidemiological data should be analyzed and compared, leading to a more comprehensive data analysis of the infection of clinically important strains. Further research on specific drug-resistant clones and the regulation of virulence genes is also needed to provide a basis for infection control and more reliable target for the development of new vaccines for the prevention of GBS invasive infections.

## Author contributions

HJ, MC, YG, and ML conceived and designed the experiments, performed all experiments and analyzed the data. TL and HL assisted in antimicrobial suscibility testing. HJ and ML supervised the study and wrote the paper. All authors read and approved the final manuscript.

## Funding

This study was supported by grants from the National Natural Science Foundation of China (grants 81322025 and 81371875), Science and Technology Committee Plan of Shanghai (14140901000), Shanghai Shuguang Talent Project (12SG03), Natural Science Foundation of Shanghai (16ZR1433300), and the Fourth 3-year Action Plan for Public Health of Shanghai Municipal Commission of Health and Family Planning (grants GWTD2015S01 and 15GWZK0101).

### Conflict of interest statement

The authors declare that the research was conducted in the absence of any commercial or financial relationships that could be construed as a potential conflict of interest.
