# Supplementary material for: Molecular Characterization of Streptococcus agalactiae Causing Community- and Hospital-Acquired Infections in Shanghai, China
Source: Front Microbiol. 2016 Aug 30;7:1308. doi: 10.3389/fmicb.2016.01308 (PMC5003847; doi:10.3389/fmicb.2016.01308)
Supplement: Supplementary file 1 [file Table1.doc]

**Molecular Characterization of *Streptococcus agalactiae* Causing Community- and Hospital-acquired Infections in Shanghai, China**

*Haoqin Jiang^1†^, Mingliang Chen^2,3†^, Tianming Li^4^, Hong Liu^1^, Ye Gong^5*^, and Min Li^4*^*

*Correspondence:

Ye Gong

[drgongye@163.com](mailto:drgongye@163.com)

Min Li

[ruth_limin@126.com](mailto:ruth_limin@126.com)

**Table S1. Sequences of primers used to identify putative and known GBS virulence genes with systematic gene names in two published reference genomes**

| Category | Gene | Function/annotation | NEM316 (III)/ A909 (Ia) | Primer sequence (5’-3’)^a^ | Size of the PCR  product (bp) |
| --- | --- | --- | --- | --- | --- |
| Adhesins | *fbsA* | fibrinogen-binding protein FbsA | GBS1087 | F: ATCAAGTCCTGTATCTGCTAT | 890 |
|  |  |  |  | R: GTGGATTTTTCCCTATAAGTTG |  |
|  | *fbsB* | fibrinogen-binding protein FbsB | GBS0850 | F: GCGCAAACTTCTGTCCAACA | 543 |
|  |  |  |  | R: TCGCCTTGATAGCAGTGTCTAC |  |
|  | *pavA* | fibronectin-binding protein | GBS1263 | F: CCTATCGTACTATCCTTCCTGG | 408 |
|  |  |  |  | R: TCTCTAACTCACTTTGAACAC |  |
|  | *scpB^b^* | C5a peptidase | GBS1308 | F: TGCCATTGCGCTTATGTCTACGAGC | 574 |
|  |  |  |  | R: TCCTGACAAGATTCCTGACACGT |  |
|  | *lmb* | laminin-binding protein | GBS1307 | F: GATGTGTTTGAAGCGTCAAAACC | 486 |
|  |  |  |  | R: TGATTTCGCAATAGCATGAGC |  |
|  | *GBS pilus cluster* | streptococcal pilus cluster | GBS0628 | F: CTCAACAGAAACACAAACACTA | 483 |
|  |  |  |  | R: CGACGCTTCATCCCCTTAACA |  |
|  |  |  | GBS0629 | F: AGGCGCAGCAGGTTTTTGAAG | 463 |
|  |  |  |  | R: GATTTTTCAATAGTCACAGC |  |
|  |  |  | GBS0630 | F: AGACCCCTACTCTGCTGAACA | 490 |
|  |  |  |  | R: ATCTTTCTGCACTGTTTTTTC |  |
|  |  |  | GBS0631 | F: TAAGCAAAAAGAAGGTTTGA | 465 |
|  |  |  |  | R: TCCGCCTCGGTAGAATCATAA |  |
|  |  |  | GBS0632 | F: CCAACACTCCCAAACGCCCACCAGG | 523 |
|  |  |  |  | R: TCCCTTCTAGATGACCAACTCC |  |
| Invasins | *cylX* | β-hemolysin/cytolysin, cyl^c^ | GBS0644 | F: TATTTTATTTTCCAACAGTA | 410 |
|  |  |  |  | R: TTGACTGAACATTACCCTG |  |
|  | *cylD* |  | GBS0645 | F: GCTGATGGCAGTGTCGTTGATTA | 855 |
|  |  |  |  | R: ACCTATTAGGAGAAATATCAAT |  |
|  | *cylG* |  | GBS0646 | F: AGAGGGGGAGGAAACAGTTATG | 830 |
|  |  |  |  | R: ATCCAACATTAAAGAGTCAAT |  |
|  | *acpC* |  | GBS0647 | F: TAGCAAGTGAGAAGGCAAAC | 416 |
|  |  |  |  | R: CCATTAGAAACGGATAGCGAT |  |
|  | *cylZ* |  | GBS0648 | F: TGTTGAACTAAATGATGATGA | 635 |
|  |  |  |  | R: CCCTCTTCAAAGATGAGATT |  |
|  | *cylA* |  | GBS0649 | F: GGTTGCCTCAGGAAGGATGT | 895 |
|  |  |  |  | R: TCAACATTAAATGTCTCAATAAT |  |
|  | *cylB* |  | GBS0650 | F: GCGTATCTCAACTTGCTTAGA | 855 |
|  |  |  |  | R: TAAATCAACATAAAGTGATTAC |  |
|  | *cylE* |  | GBS0651 | F: CACTGCCAAGAGCAGTTGATT | 558 |
|  |  |  |  | R: TTCCCCTTGGCGGATTTGGA |  |
|  | *cylF* |  | GBS0652 | F: GGCTATCAATTTTTCCTACC | 511 |
|  |  |  |  | R: CCACTAACATATACGCTCAT |  |
|  | *cylI* |  | GBS0653 | F: AGGGACCTCACTTGGGGGAA | 581 |
|  |  |  |  | R: CATTAGCTTGAGTACCTGTA |  |
|  | *cylJ* |  | GBS0654 | F: GGTGGTATCTCTTTTGCTATTA | 809 |
|  |  |  |  | R: ATTCCACCCAATCTCGTTTAA |  |
|  | *cylK* |  | GBS0655 | F: TCTTGAAAGAGGCTATGATA | 764 |
|  |  |  |  | R: TAAATTCCTGCAAAGGTATTA |  |
|  | *cfb* | CAMP factor | GBS2000 | F: TAGGGGAAAAGAAAGCGCTTGA | 614 |
|  |  |  |  | R: GCCTTTACATCGTTAACTTGA |  |
|  | *spb1* | hemolysin III | GBS1477 | F: CCGAAAAGACAAAGGTGTTGT | 450 |
|  |  |  |  | R: CCATCTACTGCCCAGTCTTTAAT |  |
|  | *hylB* | hyaluronate lyase | GBS1270 | F: GATATAGAAGGCTCTGCCAA | 413 |
|  |  |  |  | R: GGCAGCAATTGTGTCAAACCA |  |
|  | *rib* | surface protein rib | GBS0470 | F: CACCAGAGGCTAAGCATAGGA | 496 |
|  |  |  |  | R: ACAGTTTGATCTTTACCTGC |  |
|  | *bca* | C-α protein | SAK0517 | F: TAACAGTTATGATACTTCACAGAC | 535 |
|  |  |  |  | R: ACGACTTTCTTCCGTCCACTTAGG |  |
| Immune evasins | *bac* | C-β protein | SAK0186 | F: TCCTTGCCTCCTTTAGAAAGA | 519 |
|  |  |  |  | R: AGCCCCAGATACACCGCAGGCT |  |
|  | *cpsM* | cps gene cluster | GBS1237 | F: ATTCAGGGAGTGCCTTTTCTG | 901 |
|  |  |  |  | R: GCGCCATAGGCTGCATAAT |  |
|  | *cpsIaJ* |  | GBS1238 | F: AGTAGCTGGAGAAATTGGGA | 766 |
|  |  |  |  | R: CGCTCGTCTAATGCTGATAAAAC |  |
|  | *cpsJ* |  | GBS1239 | F: TAGATCCTAATCATTGGAGTA | 880 |
|  |  |  |  | R: GATTTTATTATCAATATTACT |  |
|  | *cpsI* |  | GBS1240 | F: TCAGGGAAATAATGATATG | 881 |
|  |  |  |  | R: TGATAAACTATCATCCTAGTG |  |
|  | *cpsG* |  | GBS1241 | F: GTATCCTGTAACAGATAAAT | 635 |
|  |  |  |  | R: ATACCATTGGTTTGGTACT |  |
|  | *cpsF* |  | GBS1242 | F: ATTAAGATTATTCTCCTAAC | 600 |
|  |  |  |  | R: TTTAATCTATCAACTTCTTTA |  |
|  | *cpsE* |  | GBS1243 | F: AGCGATGATTCAAACAGTTGT | 909 |
|  |  |  |  | R: ATCCTACCATTACGACCTAC |  |
|  | *cpsD* |  | GBS1244 | F: GATGATCGTGTTAATACTGAA | 808 |
|  |  |  |  | R: TACAAGAGCCCCTTACTTCC |  |
|  | *cpsC* |  | GBS1245 | F: AGCAGAAGCTTATAAGATTAT | 907 |
|  |  |  |  | R: ACTAAACTGTATGTTTGTAC |  |
|  | *cpsB* |  | GBS1246 | F: ACGGGTGAACTCCCTTCATAT | 958 |
|  |  |  |  | R: TGCAATGGCAACAAATGTGA |  |
|  | *cpsA* |  | GBS1247 | F: ACTAATCATAAAGAAGGGGTAT | 862 |
|  |  |  |  | R: CTCTCTTCAAGCGTTTTAGG |  |
|  | *neuA* | neu gene cluster | GBS1233 | F: TCCTGCGCGATCAGGGTCAA | 870 |
|  |  |  |  | R: GAAACAAAAGCCTCTGTTACA |  |
|  | *neuD* |  | GBS1234 | F: CCTACTACAAAGGGAATGCTT | 840 |
|  |  |  |  | R: TCATGGGTTTACCTGCTAGGA |  |
|  | *neuC* |  | GBS1235 | F: AGGGGATCGTTACGAAATGT | 463 |
|  |  |  |  | R: AGCCTTTGCAATATCATCAGA |  |
|  | *neuB* |  | GBS1236 | F: AGATTGGCAAGCAACAAAAG | 660 |
|  |  |  |  | R: CGATTATAATTAGGTGTTATTA |  |
|  | *cspA* | serine protease cspA | GBS2008 | F: ACAGCGTTTTTCAATCCGGA | 486 |
|  |  |  |  | R: CAACAGTTCCCTCTCCTTTGT |  |
|  | *pbp1A* | penicillin-binding protein 1A | GBS0288 | F: CAGACCGCGATTGGGGTTCTA | 552 |
|  |  |  |  | R: CTGCTTTAGTACCAGTACCA |  |

a. F, forward; R, reverse.

b. dual roles of both an adhesin and an immune system evading gene.

c. although primarily an invasin, cyl is capable of damaging phagocytes and hence also have a role in immune system evasion
